# Strictinin, a novel ROR1-inhibitor, represses triple negative breast cancer survival and migration via modulation of PI3K/AKT/GSK3ß activity

**DOI:** 10.1371/journal.pone.0217789

**Published:** 2019-05-31

**Authors:** Norman Fultang, Abhinav Illendula, Brian Chen, Chun Wu, Subash Jonnalagadda, Nathan Baird, Zachary Klase, Bela Peethambaran

**Affiliations:** 1 Department of Biology, University of the Sciences in Philadelphia, Philadelphia, Pennsylvania, United States of America; 2 Department of Chemistry & Biochemistry, Rowan University, Glassboro, New Jersey, United States of America; 3 Department of Chemistry & Biochemistry, University of the Sciences in Philadelphia, Philadelphia, Pennsylvania, United States of America; King Faisal Specialist Hospital and Research Center, SAUDI ARABIA

## Abstract

Triple Negative Breast Cancer (TNBC), the most aggressive subtype of breast cancer, is characterized by the absence of hormone receptors usually targeted by hormone therapies like Tamoxifen. Because therapy success and survival rates for TNBC lag far behind other breast cancer subtypes, there is significant interest in developing novel anti-TNBC agents that can target TNBC specifically, with minimal effects on non-malignant tissue. To this aim, our study describes the anti-TNBC effect of strictinin, an ellagitanin previously isolated from *Myrothamnus flabellifolius*. Using various in silico and molecular techniques, we characterized the mechanism of action of strictinin in TNBC. Our results suggest strictinin interacts strongly with Receptor Tyrosine Kinase Orphan like 1 (ROR1). ROR1 is an oncofetal receptor highly expressed during development but not in normal adult tissue. It is highly expressed in several human malignancies however, owing to its numerous pro-tumor functions. Via its interaction and inhibition of ROR1, strictinin reduced AKT phosphorylation on ser-473, inhibiting downstream phosphorylation and inhibition of GSK3β. The reduction in AKT phosphorylation also correlated with decreased cell survival and activation of the caspase-mediated intrinsic apoptotic cascade. Strictinin treatment also repressed cell migration and invasion in a beta-catenin independent manner, presumably via the reactivated GSK3ß’s repressing effect on microtubule polymerization and focal adhesion turnover. This could be of potential therapeutic interest considering heightened interest in ROR1 and other receptor tyrosine kinases as targets for development of anti-cancer agents. Further studies are needed to validate these findings in other ROR1-expressing malignancies but also in more systemic models of TNBC. Our findings do however underline the potential of strictinin and other ROR1-targeting agents as therapeutic tools to reduce TNBC proliferation, survival and motility.

## Introduction

Approximately one in eight women are diagnosed with breast cancer each year. Of these new cases, 15–20% are Triple Negative [1]. Triple Negative Breast Cancer (TNBC) is an aggressive subtype of breast cancer characterized by malignant cells void of hormone (Progesterone and Estrogen) receptors and Human Epidermal Growth Factor Receptor 2 (HER-2) [2]. The lack of these receptors, usually targeted by hormonal therapies like tamoxifen and fulvestrant, leads to an increased difficulty in treatment. Owing to its increased propensity for metastasis and invasion, TNBC prognosis and long-term survival is significantly worse than in other breast cancer subtypes [3]. In addition, traditional chemotherapeutics and radiation used in breast cancer therapy are often highly toxic to both TNBC cells and healthy cells. This leads to many negative and unwanted side effects, often causing a decrease in therapy adherence in patients. In recent years, there has thus been an increased effort to look for novel agents that can treat triple negative breast cancer or complement existing drugs, with minimal effects on non-malignant cells.

Phyto-compounds with anti-cancer activity that can selectively target TNBC with minimal effects on non-malignant cells have emerged as viable therapeutic agents for TNBC. We previously isolated strictinin (Chemical name: 3-O-galloyl-4,6-[(S)- hexahydroxydiphenoyl]-b-d-glucopyranose), from *Myrothamnus flabellifolius*, a South African medicinal plant with anti-cancer properties [4,5]. Strictinin was shown to have pronounced anti-TNBC activity with minimal activity on the non-malignant MCF-10A breast epithelial line.

To identify the molecular targets of strictinin in TNBC, we used its structure to search drugs in DrugBank [6] with 0.75 of structure similarity threshold, one hit with 0.795 of similarity score was obtained: an experimental drug named 1,2,3,4,6-Pentagalloyl glucose (DB03208). Although the targets of DB03208 were not reported in DrugBank, a recent in-silico drug discovery study [7] suggest DB03208 is a potential cancer drug by inhibiting the activity of Receptor Orphan Tyrosine Kinase-like Receptor-1 (ROR1), an oncofetal receptor present during embryonic development but absent or expressed minimally in normal adult tissue. It is however highly expressed in several human malignancies including breast, ovarian, lung, lymphoma, skin, pancreatic, testicular, uterus, prostate and adrenal cancers [8,9]. Therefore, we hypothesized that strictinin acts as an ROR1 inhibitor just like DB03208.

The ROR1 protein is a member of the RTK-family of tyrosine kinase receptors but interestingly lacks any significant kinase activity [10]. Structurally, it contains an extracellular Ig-like domain, a cysteine-rich domain, a Frizzled domain, a kringle domain, a transmembrane domain with tyrosine kinase, serine/threonine-rich and proline-rich domains on the cytoplasmic side [11]. Functionally, upon binding of its ligand (Wnt3/5a), it interacts with the non-canonical Wnt signaling pathway, activating Wnt-responsive genes in a beta-catenin independent manner [12,13]. This culminates in EMT-induction and proliferation [14]. It also promotes AKT phosphorylation at serine-473 potentially through its interaction with casein kinase1ε (CK1ε) [8] resulting in increased cell survival, proliferation and migration. It has emerged in recent years as a target of significant interest for immunotherapy and drug discovery [15–17].

Here we report that strictinin inhibits ROR1 activity, reducing downstream phosphorylation and activation of AKT by P13K on serine-473. This coincided with reduced TNBC survival and induction of intrinsic apoptosis via inhibition of Bad phosphorylation and cleavage of caspase 9. We also observed a reduction in GSK3ß phosphorylation at serine-9 by p-AKT, a process which inhibits its activity. The newly “re-activated” GSK3ß, presumably via its action on microtubule dynamics and maturation of nascent focal adhesions repressed TNBC cell motility in a beta-catenin independent manner. Our results describe the anti-proliferative and anti-metastatic potential of strictinin as an anti-TNBC agent and underlines the intersection of the ROR1/P13K/AKT/GSK3ß pathway as a target for the development of “multi-front” cancer therapies.

## Materials and methods

### Cell culture

TNBC lines MDA-MB231 (ATCC HTB 26), BT549 (ATCC HTB122) and Normal Human Breast Epithelial cell line (MCF-10A) (ATCC CRL-10317) are cultured as per ATCC recommendations. MDA-MB-231cells were cultured in DMEM/F-12 (Lonza, Basel, Switzerland) supplemented with FBS (10%), insulin (10μg/ml), non-essential amino acids, sodium pyruvate, penicillin (100 U/ml) and streptomycin (0.1 mg/ml). BT-549 cells were cultured in RPMI (Lonza, Basel, Switzerland), with FBS (10%), insulin (10μg/ml), and penicillin (100 U/ml) and streptomycin (0.1 mg/ml). MCF-10A was grown in DMEM/F-12 with 5% horse serum (ATCC, Manassas, VA, USA), insulin (10μg/ml), hydrocortisone (0.5 mg/ml), EGF (20ng/ml), penicillin (100 U/ml) and streptomycin (0.1 mg/ml). All cells were kept at 37°C with 5% CO_2_.

### MTT assay

MTT assays (Thermofisher, Waltham, MA, USA) were used to assess proliferation and viability. Cells were seeded in a 96-well plate at a density of 25,000 cells/well. Strictinin, dissolved in DMSO (final DMSO concentration < 0.3%) was added to the cells. A 0.3% DMSO vehicle control group was also included in all analyses. After 24-72h, treatment media was replaced with fresh media and MTT dye added. Following a 2-hr incubation at 37°C, DMSO was added and absorbance read at 540 nm. Cell viability was calculated as percentage of no-treatment control.

### *In silico* docking analysis

#### Ligand and protein structure

**Fig 1:**
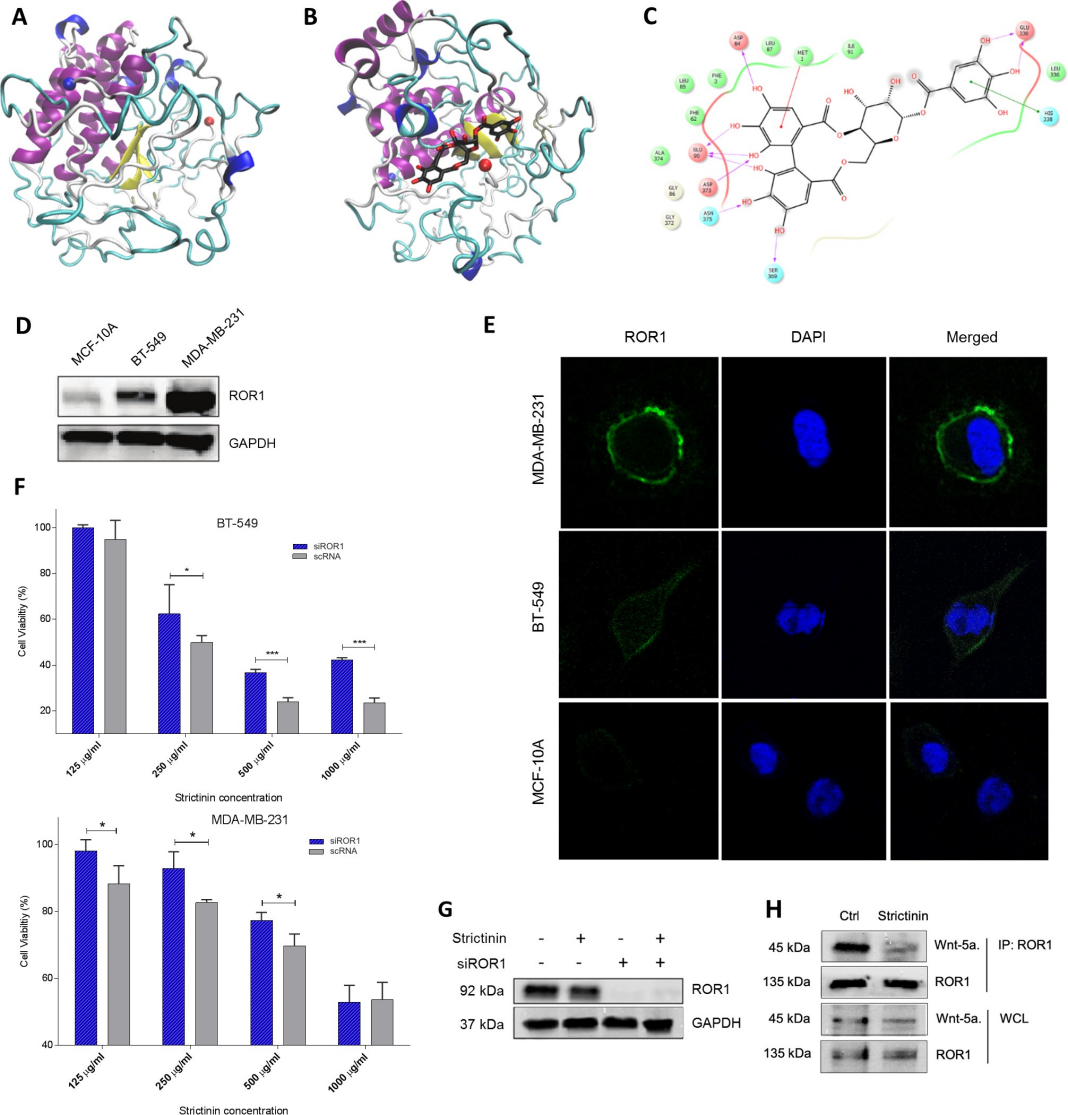
Strictinin interacts with ROR1 in TNBC. **a)** Homology Model of tROR1. Whereas N-terminus is indicated by a red ball, C-terminus is indicated by a blue ball. **b, c)** Strictinin docked to tROR1 and ligand interactions diagram. Whereas N-terminus is indicated by a red ball, C-terminus is indicated by a blue ball. d, **e)** Immunoblot and immunofluorescence investigating basal ROR1 expression in two TNBC lines and non-malignant breast epithelial line, MCF-10A. **f)** MTT assessing effect of ROR1 siRNA knockdown on strictinin cytotoxicity in TNBC. **g)** Immunoblot showing effect of siROR1 knockdown and strictinin on ROR1 expression in MDA-MB-231. h) Co-immunoprecipitation of ROR1 receptor showing inhibition of Wnt-5a binding to ROR1 after strictinin treatment. (* = p.value <0.05, n = 3).

3D structures of the ligands, DB03208 and strictinin, were built in Schrodinger’s Maestro. For each ligand, the tautomeric states were generated at pH = 7 using Maestro’s Epik [18,19]. The lowest tautomeric state was selected and then minimized to the most energetically favorable structure. Receptor Tyrosine Kinase-like Orphan Receptor 1 (ROR1) has an extracellular domain, a transmembrane domain, and an intracellular domain. We modeled a truncated version of ROR1 (tROR1) that is identical with the intracellular, C-terminal region of ROR1 but does not contain the transmembrane nor the extracellular domains. The sequence for tROR1 (AAC50714.1) was retrieved from the NCBI protein database. The 3D structure of tROR1 (Fig 1) was then predicted using I-TASSER [20–22]. The model for tROR1 was then preprocessed, optimized, and minimized using Maestro’s Protein Preparation Wizard [23]. We then mapped for active sites using Maestro’s SiteMap [24,25].

#### Ligand docking

The active site (S1A Fig) we chose was the one closest to the residues indicated in a previous docking of DB03208 to tROR1 by Nath et Al [7]. A receptor grid was generated around the active site of tROR1. DB03208 and Strictinin were individually docked to tROR1 using Glide docking followed by Induced Fit Docking [26–31]. To check the validity of our docking, we compared our ligand interaction diagram to that of Nath et Al [7]. Our DB03208 ligand interactions showed many similar residue interactions to theirs (S1B and S1C Fig). The binding site of strictinin is very close to the binding site of DB03208 (S1D Fig), suggesting these two ligands might have similar drug action against tROR1.

### Immunoblotting

Following various treatments, the cells were washed once in 1X PBS and lysed with RIPA buffer (150 mM NaCl, 1.0% Triton-X-100, 0.5% Sodium deoxycholate, 0.1% SDS, 50 mM Tris, pH 8.0) with 1 mM PMSF. Protein concentrations were determined by Bradford Assay using Pierce 660 nm Protein Assay reagent (thermofisher, Waltham, MA, USA). Following SDS-PAGE, Proteins were transferred to nitrocellulose membranes, which were blocked for 1-hour in 5% milk with gentle agitation. Membranes were incubated overnight at 4°C with various antibodies then washed in TBS-T (20mM Tris, 150mM NaCl, 0.1% Tween 20), and incubated for 1-hour at room temperature with horseradish-peroxidase-linked anti-Mouse or anti-Rabbit IgGs (Cell Signaling, Danvers, MA, USA). Protein bands were detected by chemiluminescence. Antibodies: ROR1 (Cell signal, #D6T8C), GAPDH (Cell signal, #D16H11), CK1ε (Cell signal, #12448S), p-AKT-ser473 (Cell signal, #9271S), AKT (Cell signal, #C67E7), p-GSK3β-ser9 (Cell signal, #D17D2), GSK3β (Cell signal, #D7SD3), XIAP (Cell signal, #D2Z8UL), caspase-9 (Cell signal, #9502S), Bad (Cell signal, #D24A9), p-Bad-ser136 (Cell signal, #4366T), β-catenin (Invitrogen, MA1-301), p-β-catenin-ser33/37, Wint-5a (Novus-Bio NBP2-24752SS)

### Co-immunoprecipitation

Co-immunoprecipitation of ROR1 and its interacting proteins was performed using the Pierce Classic IP Kit (thermofisher, # 26146) according to manufacturer protocol. Briefly, following treatments, cells were lysed in a moderate-strength IP-Lysis buffer without SDS (thermofisher, #87787) and protein quantified by Bradford assay. For each condition, 1μg of protein was incubated with Rabbit IgG anti-ROR1 ab (Cell signal, #D6T8C) at 1:100 (final volume 300 μl in IP-lysis buffer) on a nutator overnight at 4°C. The ROR1 immune complex was captured by centrifugation in a Protein A/G agarose-resin column and eluted by centrifugation using non-reducing sample buffer with DTT. Following boiling, the eluted samples were analyzed by western blot as previously described.

### Immunofluorescence

Cells were plated at a density of 3000 cells/well in wells of Lab-Tek chamber slides (Sigma-Aldrich, St. Louis, MO, USA) and fixed with pre-chilled methanol for 5 minutes at -20°C. Following three PBS rinses, cells were incubated overnight in primary antibody diluted in 5% BSA/PBS. Cells were once again rinsed with PBS and incubated at 37°C with Alexa-fluor-555 (Thermofisher, Waltham, MA, USA) secondary antibody in 5% BSA/PBS for an hour. Following three PBS washes, cells were incubated in DAPI stain solution (0.2μg/ml in PBS) for 2 minutes at room temperature. After an additional 3 PBS washes, the cells were imaged via confocal microscopy on a Leica DMi8 Microscope (Leica, Wetzlar, Germany).

### Foxo luciferase assay

P13K/AKT activity was assessed using a FOXO luciferase reporter (BPS bioscience, #60643, San Diego, CA, USA) according to manufacturer's protocol. Briefly, TNBC cells were transfected with a construct encoding a firefly luciferase gene under the control of FOXO responsive elements which are targets of the P13K/AKT pathway. Following treatments, cells were lysed and luciferase activity assessed using Promega’s Dual-Luciferase Reporter Assay System (Promega, E1910, Madison, WI, USA).

### RNAi knockdown of ROR1

ROR1-siRNA (Thermofisher, AM16708) was transfected into cells using Lipofectamine RNAi/Max reagent (Thermofisher, Waltham, MA, USA) according to manufacturer protocol. Briefly, cells were transfected with siROR1 for 24h in Opti-MEM (Thermofisher, Waltham, MA, USA) then rescued in full culture media for 2 hours before treatment or lysing for protein.

Annexin-V/FITC staining: Annexin-V/FITC staining to assess apoptosis after Strictinin treatment was performed using the Muse Annexin V and Dead Cell Assay Kit (Emdmillipore, MCH100105, Burlington, MA, USA) according to the manufacturer’s protocol. Briefly, treated cells were trypsinized and re-suspended in fresh culture media before addition of Annexin-V/FITC dye solution. Stained cells were analyzed for apoptosis by FACS using a Muse Cell Analyzer.

### Wound healing assay

Wound healing assays to assess cell migration were conducted in 24-well plates. 150,000 cells were seeded in each well and allowed to propagate for 24h to get a confluent monolayer. Cells were then treated with appropriate treatments for 24h. A 10μl pipette tip was used to make a wound on the monolayer and an inverted phase contrast microscope was used to visualize and take pictures of the wound. Quantification of wound size was done on ImageJ and percent migrated calculated as percentage of wound closed.

### Invasion assay

Boyden chamber assays to assess cell invasion were conducted in 24-well plates with a Cultrex 24 Insert Cell Invasion / Migration Chamber (Trevigen, 3455-024-01, Gaithersburg, MD, USA). Cells were serum starved for 24h before being seeded in the top chamber of the transwell set-up. Following treatment, complete culture media containing FBS was added to the bottom chamber. At varying time points, both complete culture media and cell solution were aspirated off and the membrane washed with PBS before crystal violet staining and phase contrast imaging. Quantification of migrated cells was done using ImageJ.

### Isothermal titration calorimetry

Binding affinity of Strictinin to ROR1 was conducted using an Affinity ITC (TA Instruments). Recombinant ROR1 (Peprotech, 160–04) was prepared in (0.1% DMSO in molecular grade water)to a final concentration of 13.8 μM and placed in the ITC cell. Strictinin was prepared in the identical buffer to a final concentration of 276 μM and placed in the syringe. Samples were loaded manually and data were collected at 37°C. Data were analyzed using NanoAnalyze v3.8.0 (TA Instruments) and Origin 2016 (OriginLab) was used to prepare the figure.

## Results

### Strictinin interacts with ROR1 in TNBC

To support our hypothesis that strictinin can interact with ROR1 in a similar way as DB03008, we did *in silico* docking analyses of DB03208 and strictinin with ROR1. Induced fit docking for DB03208 and strictinin to the similar site resulted in a tight binding for both ligands (Fig 1B and 1C). DB03208 had a Glide gscore of -11.4 kcal/mol while strictinin had a Glide gscore of -7.7 kcal/mol (Table 1). MM-GBSA calculations were also run resulting in -116.8 kcal/mol for DB03208 and -97.8 kcal/mol for strictinin. Given the moderate binding affinity at the similar binding site of DB03208, it is plausible that strictinin interact with ROR1 just like DB03208. The detailed ligand-protein interaction for strictinin is shown in Fig 1.

**Table 1:** Glide scores of DB03208 and Rx7.

Ligand	Glide gscore (kcal/mol)	MMGBSA (kcal/mol)
DB03208	-11.4	-116.8
Rx7	-7.7	-97.8

To experimentally evaluate whether strictinin binds ROR1, we next employed isothermal titration calorimetry. Indeed, the binding isotherm indicates a direct binding interaction with low micromolar affinity (S1E Fig). Strictinin binding is enthalpically driven and confirms a one-to-one binding stoichiometry with ROR1. To validate our *in silico* and ITC findings, we set out to knockdown ROR1 expression in TNBC cell lines and observe the effect on strictinin cytotoxicity. First we assessed basal ROR1 expression in our two TNBC cell lines (MDA-MB-231 and BT-549) and one non-malignant cell line (MCF-10A). We found high expression of ROR1 in both TNBC cell lines with minimal expression in the non-malignant cell line MCF-10A (Fig 1D and 1E). We then conducted cell viability assays after siRNA knockdown of ROR1 in both TNBC cell lines and observed repression of strictinin cytotoxicity (Fig 1F and 1G). Further corroborating our results that strictinin interacts with ROR1, we also observed inhibition of Wnt-5a binding to ROR1 after treatment with strictinin (Fig 1H). Altogether, the ITC, co-immunoprecipitation, and the siROR1 cell viability data provide evidence that strictinin binds competitively to ROR1.

### Strictinin inhibits AKT phosphorylation and GSK3ß inhibition

**Fig 2:**
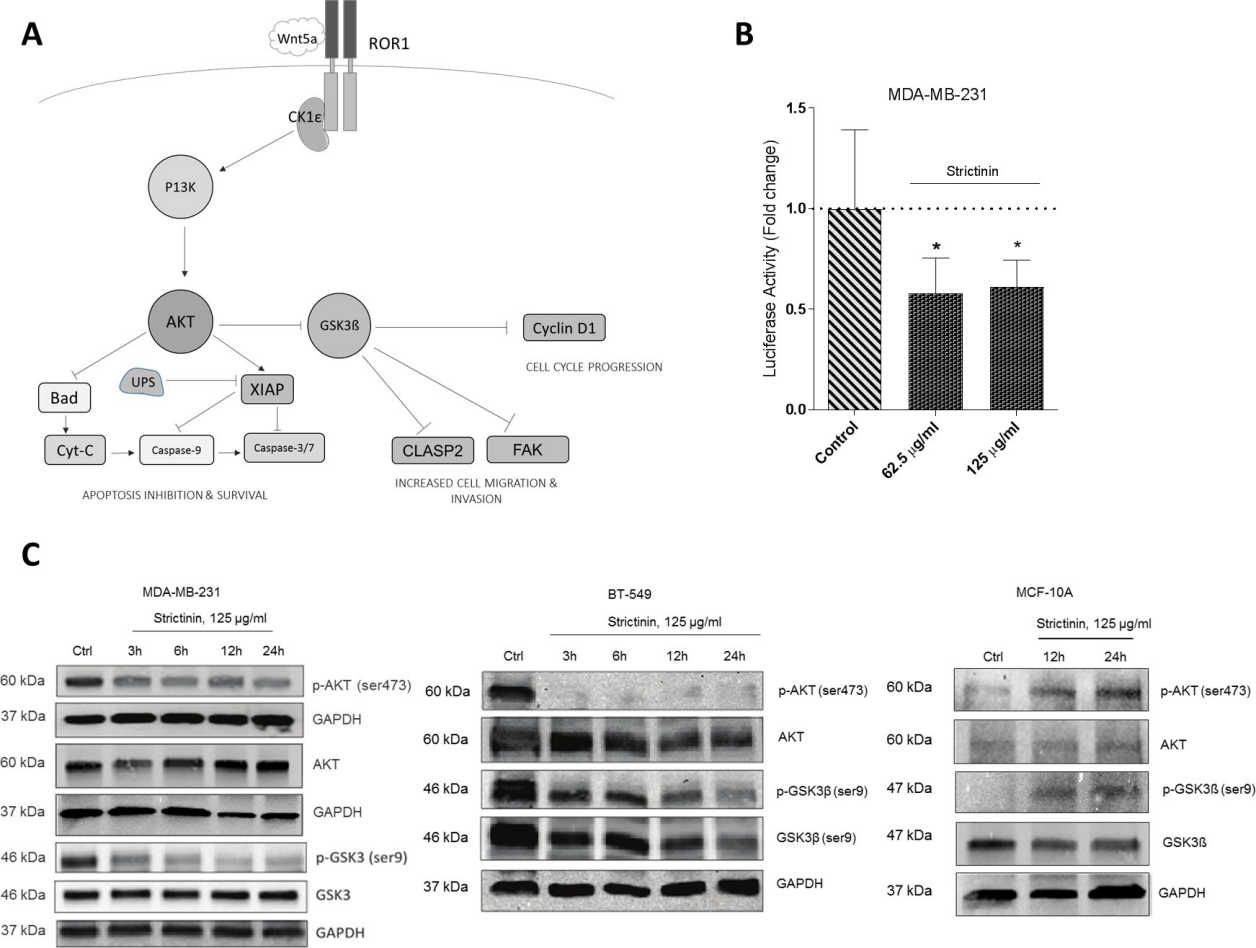
Strictinin inhibits AKT phosphorylation and downstream GSK3ß phosphorylation. **a)** The ROR1 signaling network as currently understood. **b)** FOXO-luciferase assay assessing srtictinin effect on P13K/AKT activity in MDA-MB-231 after 24h. **c)** Immunoblots assessing strictinin effect on AKT phosphorylation at serine 473 and GSK3β phosphorylation at serine 9. (* = p.value <0.05, n = 3).

The ROR1 signaling pathway culminates in activation of AKT via phosphorylation on serine-473 by P13K (Fig 2A). To investigate disruption of this phosphorylation event and the P13K/AKT pathway at large, we used a FOXO-luciferase reporter assay which directly measures activity of the P13K/AKT pathway. We observed a decrease in P13K/AKT activity in the TNBC cell lines after treatment with 62.5 and 125 μg/ml of strictinin for 24h (Fig 2B, S2A Fig). Further corroborating our results were immunoblots which showed a time-dependent decrease in p-AKT (ser9) levels after TNBC cell treatment with strictinin, an effect which was reversed in the MCF-10A line (Fig 2C). Because recruitment of CK1ε by ROR1 had been previously shown to be necessary for its stimulatory activity on P13K/AKT, we used co-immunoprecipitation to assess CK1ε binding to ROR1 after strictinin treatment. However, we observed no significant change in ROR1-bound CK1ε levels after strictinin treatment (S2B Fig).

### Strictinin induces apoptosis and reduces cell survival

**Fig 3:**
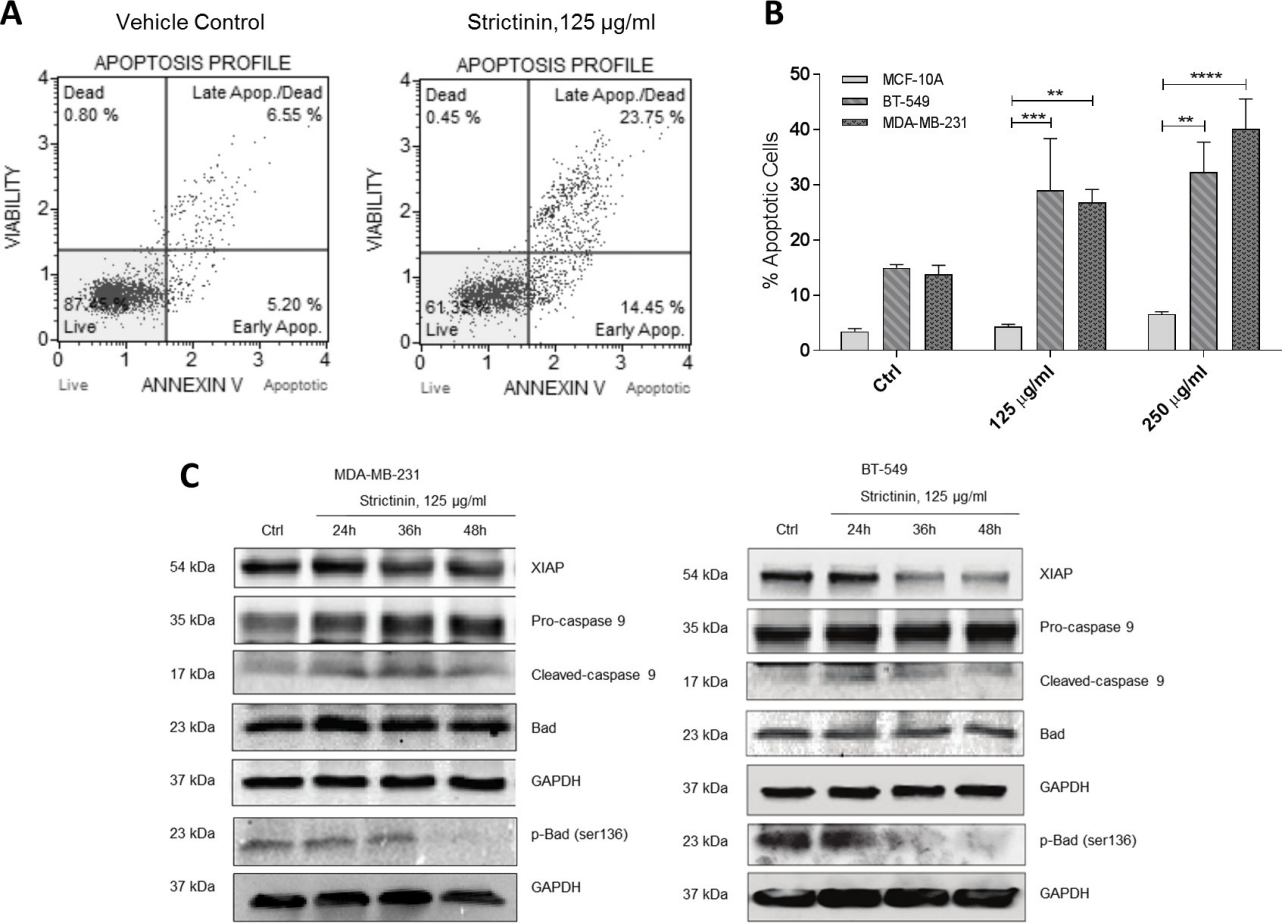
Strictinin induces apoptosis and reduces cell survival. **a, b)** Annexin-V/FITC staining and FACS to assess apoptosis in MDA-MB-231 after treatment with strictinin. **b)** Quantification of strictinin induction of apoptosis (top and bottom right quadrants) in both TNBC lines and non-malignant MCF-10A line. **c)** Immunoblots investigating modulation of pro- and anti-apoptotic proteins downstream of ROR1 after strictinin treatment.

Because ROR1 activity and activation of AKT has been shown to promote cancer cell survival and inhibit apoptosis [8], we used Annexin-V/FITC staining to assess apoptosis induction after strictinin treatment. Flow cytometric analyses of the stained cells revealed strictinin treatment induced a dose-dependent increase in apoptosis in the two TNBC cell lines but not in the MCF-10A cell line (Fig 3A and 3B). Immunoblots also revealed a decrease in XIAP levels and Bad phosphorylation on serine-136 after treatment with strictinin. This culminated in cleavage of caspase-9 and activation of the intrinsic apoptosis cascade (Fig 3C).

### Strictinin represses TNBC cell motility

**Fig 4:**
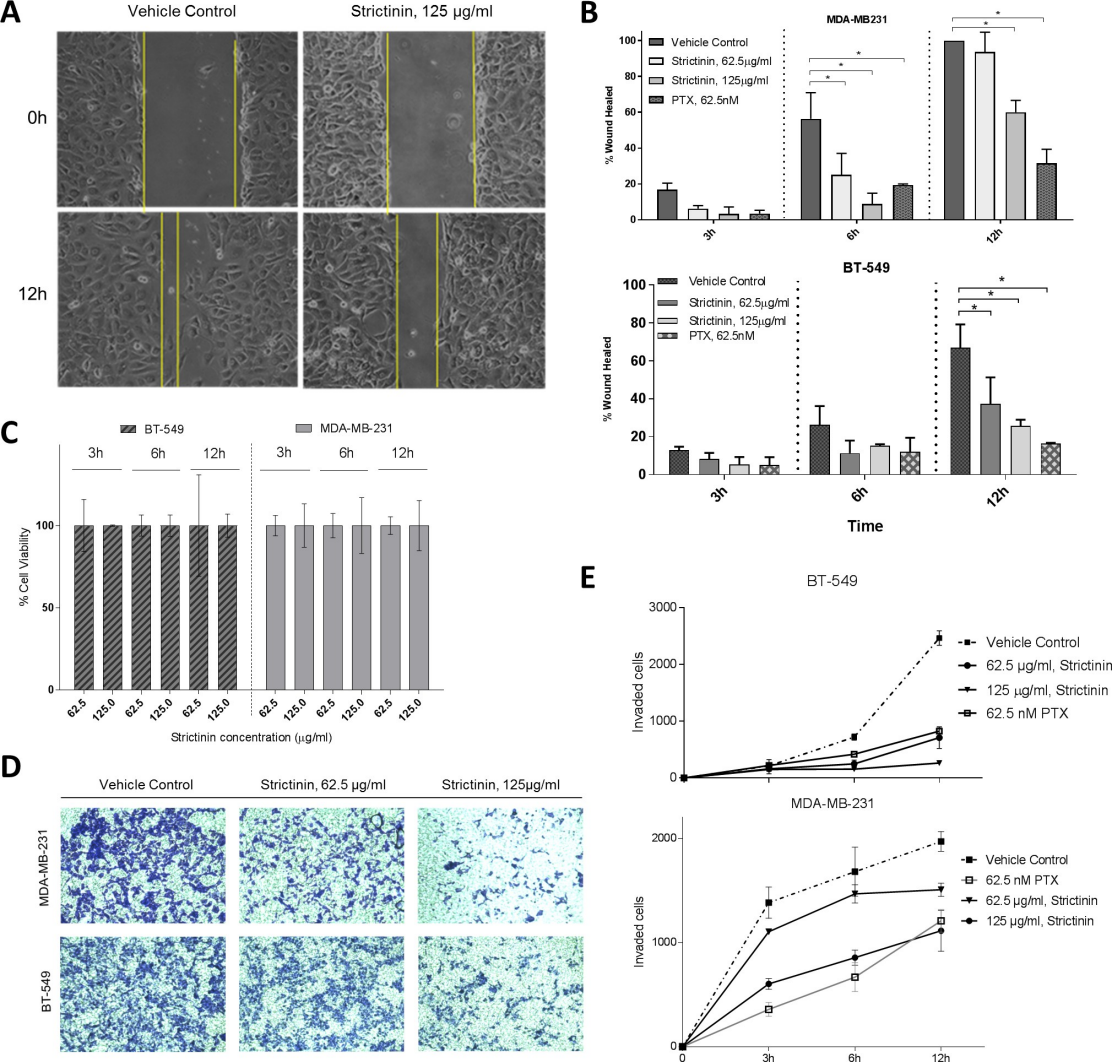
Strictinin represses TNBC cell motility. **a)** Wound healing assay investigating strictinin effect on MDA-MB-231 migration. **b)** Quantification of wound healing assays in two TNBC lines and non-malignant MCF-10A. **c)** MTT viability assay to investigate proliferation and viability during wound healing assay. **d)** Transwell assay investigating effect of strictinin on TNBC cell invasion. **e)** Quantification of transwell assay assessing strictinin effect on TNBC cell invasion. (* = p.value < 0.05, n = 3).

ROR1 has similarly been shown to promote TNBC cell motility [32,33]. We sought out to investigate if strictinin by inhibiting ROR1 activity might repress cell migration and invasion. Wound healing assays suggest strictinin treatment repressed MDA-MB-231 and BT-549 migration and invasion in a dose-dependent manner (Fig 4A, 4B, 4D and 4E). We also conducted control cell viability assays to ensure changes in cell migration weren’t as a result of proliferation and confirmed no significant change in cell viability or numbers at the strictinin doses and times assessed (3-12h, 62.5 and 125 μg/ml) (Fig 4C). MCF-10A, which are normally non-migratory, showed no significant change in migration after strictinin treatment (S3 Fig).

### Strictinin effect on cell migration is regulated via GSK3ß in a ß-catenin independent manner

**Fig 5:**
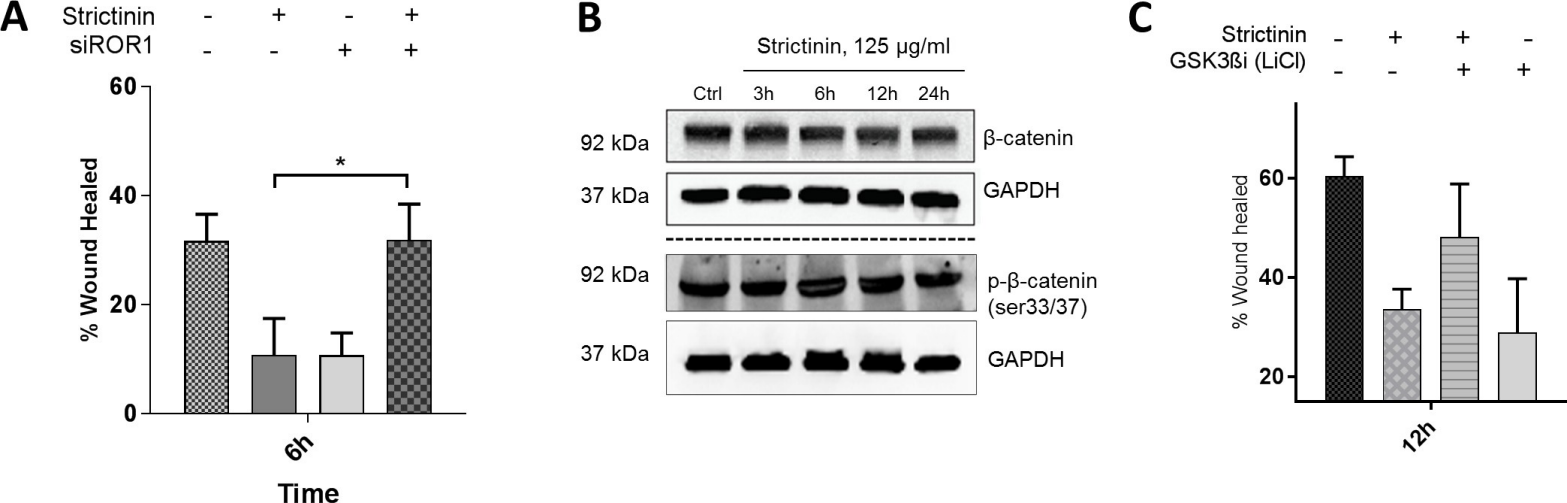
Strictinin effect on cell migration is GSK3ß and ROR1 -dependent but ß-catenin independent. **a)** Wound healing assay investigating strictinin effect on MDA-MB-231 migration after siROR1 knockdown. **b)** Immunoblot investigating beta-catenin phosphorylation and degradation by the destruction complex after treatment with strictinin. **c)** Wound healing assay investigating phenotypic rescue of migration by inhibition of GSK3ß after strictinin treatment in MDA-MB-231. (* = p.value <0.05, n = 3).

ROR1 is a non-canonical Wnt receptor which suggests it functions in a ß-catenin-independent manner but because strictinin, via its action on AKT, induced such dramatic changes in GSK3ß phosphorylation, a key member of the ß-catenin destruction complex, we opted to verify if ß-catenin was being affected in any way. Immunoblots revealed no changes in ß-catenin phosphorylation on serine-33/37, the residues usually phosphorylated by GSK3ß and the destruction complex. We also noted no significant changes in whole ß-catenin levels suggesting no increased degradation of the protein (Fig 5A). To verify the mechanism of strictinin action on migration was ROR1-dependent, we conducted siROR1 knockdown wound healing assays. Our results suggested siRNA knockdown of ROR1 repressed strictinin’s effect on TNBC migration (Fig 5B). After transfection, there was a partial rescue in cell migration even after strictinin treatment. GSK3ß is a key regulator of the cell motility and so we opted to verify if the observed repression of cell migration was GSK3ß-dependent. We treated cells with strictinin and/or LiCl, a known GSK3ß inhibitor, and monitored cell migration. We observed phenotypic rescue after treatment with LiCl and strictinin. LiCl partially repressed strictinin’s inhibitory effect on cell migration but by itself repressed cell migration.

## Discussion

Current chemotherapeutic agents for TNBC, including taxanes and anthracyclines have been shown to be both hepatotoxic and cardiotoxic to patients [34,35] leading to disastrous side effects and a decrease in therapy adherence. The development of novel targeted anti-TNBC agents that can target TNBC with minimal effects on normal tissue is thus an especially vital mission in our efforts to treat the disease. Oncofetal receptors which are minimally expressed in normal adult tissue but overexpressed in most cancers are appealing candidates for the development of these targeted agents. One such receptor is the ROR1 receptor which is highly expressed in most human malignancies but only minimally in normal tissue [9]. ROR1 has emerged recently as a molecule of significant interest for the development of targeted small molecules, antibodies, chimeric antigen T-cells and other agents for cancer therapy [15–17]. In this study we describe the mode of action of strictinin, one such targeted agent, which inhibits the action of ROR1, repressing TNBC migration and survival via P13K/AKT/GSK3ß deregulation.

To identify molecular targets for strictinin, we opted to use a non-blinded screening approach by searching DrugBank. DB03008, a molecule very similar in structure to strictinin (same glucopyranose backbone with branching galloyl subgroups), was previously shown to have high affinity for ROR1 in an *in silico* drug discovery screen for possible ROR1 antagonists [7]. It has also been shown to disrupt the activity of pathways downstream to ROR1 such as P13K/AKT in breast cancer [36]. We hypothesized that strictinin, might similarly interact with ROR1, inhibiting its function and disrupting the activity of pathways downstream to it.

We conducted a similar *in silico* docking experiment to the one performed by Nath et al [7] and our results suggested strictinin may have affinity for ROR1. We confirmed strictinin and ROR1 interaction by ITC and monitoring Wnt5a binding to ROR1 in presence of strictinin. To further validate these findings, we transiently knocked down ROR1 expression in our TNBC lines via siRNA and found a reduction in strictinin cytotoxicity in these lines. This would suggest ROR1 is a target for strictinin in TNBC and high expression of ROR1 might be partially necessary for strictinin-mediated cell death. Further corroborating these data, is the fact that the non-tumorigenic line, MCF-10A, which only minimally expresses ROR1, shows significant resistance to strictinin-induced cytotoxicity [4].

ROR1 signaling promotes breast cancer migration and survival via downstream activation of the P13K/AKT pathway [8] resulting GSK3ß inhibition. Upon binding of the Wnt5a ligand, ROR1 recruits CK1ε and promotes P13K-mediated phosphorylation of AKT on ser473. Highly active p-AKT promotes survival by inhibiting apoptosis. Through its phosphorylation of Bad on ser136, it prevents heterodimerization of Bad, Bax and Bak in the inner mitochondrial membrane to form a channel for Cytochrome C escape into the cytoplasm [37,38]. It also phosphorylates XIAP allowing it to evade the Ubiquitin-Proteasome System (UPS). Stabilized XIAP then directly inhibits the action of initiator caspase-9 and executioner caspase-3, repressing apoptosis [39]. Highly active p-AKT also phosphorylates GSK3ß on ser9, directly repressing its kinase activity [37]. GSK3ß is a negative regulator of cell migration by its repressive action on microtubule growth via phosphorylation and inhibition of CLASP. It also phosphorylates and inhibits FAK, inhibiting nascent adhesion turnover, a vital process to cell motility [40,41].

Using both immunoblots and luciferase reporter assays we confirmed inhibition of AKT phosphorylation on ser473 after strictinin treatment in both TNBC lines. This coincided with a reduction in GSK3ß phosphorylation on ser9. Expectedly, in both TNBC lines we observed an increase in apoptosis induction and decrease in cell survival after strictinin treatment. Interestingly, in the non-malignant MCF-10A line, strictinin seemed to promote AKT phosphorylation with no noticeable effect on apoptosis induction or survival. This suggests strictinin’s inhibitory effect on the P13K/AKT pathway is specific to the TNBC lines presumably due to the presence of the ROR1 receptor. Immunoblots also revealed the method of apoptosis induction was intrinsic. There was a decrease in Bad phosphorylation on ser136, presumably allowing it to heterodimerize with Bak and Bax forming a channel in the mitochondrial membrane for cytochrome-C escape. When cytochrome-C is released into the cytoplasm, it binds to Apaf-1 to form the apoptosome which cleaves caspase-9, activating it [42]. We observed an increase in caspase-9 cleavage after strictinin treatment, indicative of induction of intrinsic apoptosis.

To verify if strictinin’s inhibition of ROR1 and GSK3ß phosphorylation had any effect on cell motility, we performed wound healing and transwell assays. Our results confirmed that strictinin repressed TNBC cell migration and invasion. We performed the same experiment in the non-malignant MCF-10A line and saw no noticeable effect on cell motility. This was expected as MCF-10A are a highly sedentary cell line, which much like most mature differentiated cells, migrate only minimally. We also conducted migration experiments after siRNA knockdown of ROR1 to assess if strictinin-mediated repression of cell motility was ROR1-dependent. We observed rescue of phenotype suggesting ROR1 expression is vital to strictinin action on cell motility. Similarly to our cell survival findings, strictinin’s lack of effect on MCF-10A motility, which only minimally express ROR1, further supports this hypothesis.

GSK3ß is a key upstream regulator of cell motility so we assessed whether strictinin’s effect on motility, was GSK3ß-mediated. The rationale for this was an abundance of previous work emphasizing GSK3ß’s key role in regulating cell spreading and motility [41,43–45]. Phosphorylation of GSK3ß on ser9 has also been shown to promote cell motility by repressing GSK3ß kinase activity [46]. Using LiCl, a well-established GSK3ß inhibitor, in combination with strictinin, we observed phenotypic rescue when compared to strictinin treatment alone. This suggests strictinin’s effect on motility, in addition to being ROR1-dependent might be mediated via its action on GSK3ß. Presumably, the “newly” reactivated GSK3ß after strictinin treatment, was inhibited by LiCl, resulting in increased cell motility. Interestingly, we observed a decrease in migration in the group treated with LiCl alone when compared to a vehicle control. Repression of GSK3ß beneath basal levels can occasionally have the adverse effect of repressing cell motility. In at least one instance, GSK3ß has been shown to occasionally form a complex with cytoplasmic cyclic AMP to promote focal adhesion turnover and facilitate cell motility, a direct contrast to its usual effect on focal adhesions [45]. In such cases, repressing GSK3ß beneath basal levels could repress cell motility. Further studies are however warranted to appreciate the effects of GSK3ß inhibition on TNBC motility.

In summary, we describe the mechanism of action of strictinin, a novel ROR1-inhibitor which was previously shown to selectively target TNBC cells with minimal effects on non-malignant cells. This could be of potential therapeutic interest considering heightened interest in ROR1 and other receptor tyrosine kinases as targets for development of anti-cancer agents. Further studies are needed to validate these findings in other ROR1-expressing malignancies but also in more systemic models of TNBC. Our findings do however underline the potential of strictinin and other ROR1-targeting agents as therapeutic tools to reduce TNBC proliferation, survival and motility.

## Supporting information

S1 Fig Strictinin and DB03208 interact with ROR1 at similar binding sites. **a)** SiteMap active site of tROR1 with several key residues labelled. **b, c)** DB03208 docked to tROR1 and ligand interactions diagram. Whereas N-terminus is indicated by a red ball, C-terminus is indicated by a blue ball. **d)** Strictinin (green) and DB03208 (yellow) dockings superimposed. ITC experiment was prepared with 13.8 μM ROR1 in the ITC cell and 276 μM strictinin in the syringe. Experiment was performed at 37°C in (0.1% DMSO in molecular grade water) using the Affinity ITC (TA Instruments). Raw binding data (upper panel) were analyzed and fit (lower panel) using NanoAnalyze (TA Instruments). Binding parameters, included as an inset (upper panel), indicate an enthalpically driven 1-to-1 binding interaction.
(TIFF)

S2 Fig Strictinin inhibits AKT phosphorylation and downstream GSK3ß phosphorylation independent of CK1ε. **a)**. FOXO-luciferase assay assessing strictinin effect on P13K/AKT activity in BT-549 after 24h. (* = p.value < 0.05, n = 3) **b)** Co-Immunoprecipitation of ROR1 to assess CK1ε binding after strictinin treatment.
(TIF)

S3 Fig Strictinin does not affect non-malignant MCF-10A cell motility. Wound healing assay investigating strictinin effect on MCF-10A migration (* = p.value < 0.05, n = 3).
(TIF)
