# Supplementary figures and images for: Strictinin, a novel ROR1-inhibitor, represses triple negative breast cancer survival and migration via modulation of PI3K/AKT/GSK3ß activity
Source: PLoS One. 2019 May 31;14(5):e0217789. doi: 10.1371/journal.pone.0217789 (PMC6544296; doi:10.1371/journal.pone.0217789)

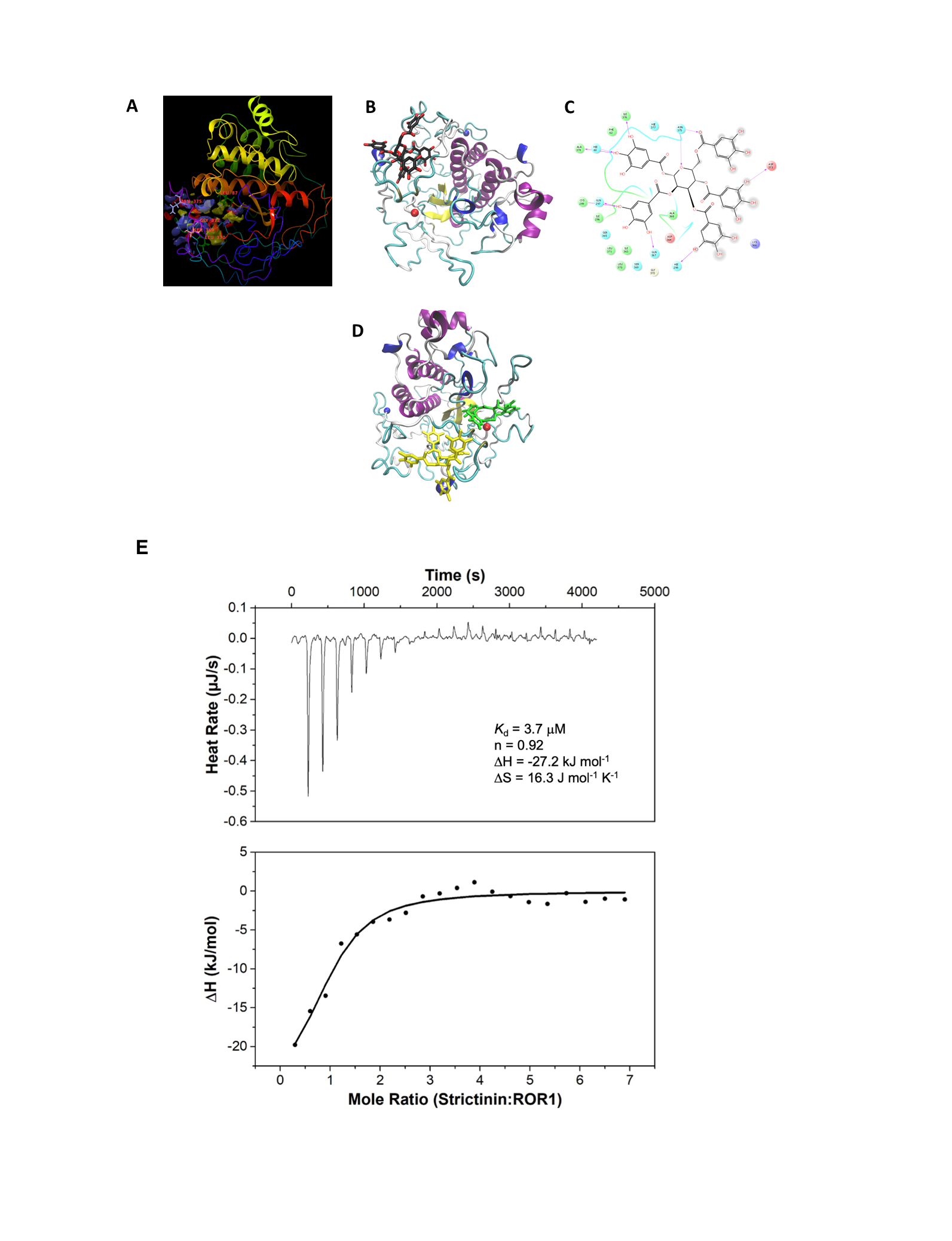

Supplement: S1 Fig — a) SiteMap active site of tROR1 with several key residues labelled. b, c) DB03208 docked to tROR1 and ligand interactions diagram. Whereas N-terminus is indicated by a red ball, C-terminus is indicated by a blue ball. d) Strictinin (green) and DB03208 (yellow) dockings superimposed. ITC experiment was prepared with 13.8 μM ROR1 in the ITC cell and 276 μM strictinin in the syringe. Experiment was performed at 37°C in (0.1% DMSO in molecular grade water) using the Affinity ITC (TA Instruments). Raw binding data (upper panel) were analyzed and fit (lower panel) using NanoAnalyze (TA Instruments). Binding parameters, included as an inset (upper panel), indicate an enthalpically driven 1-to-1 binding interaction. (TIFF) [file pone.0217789.s001.tiff]

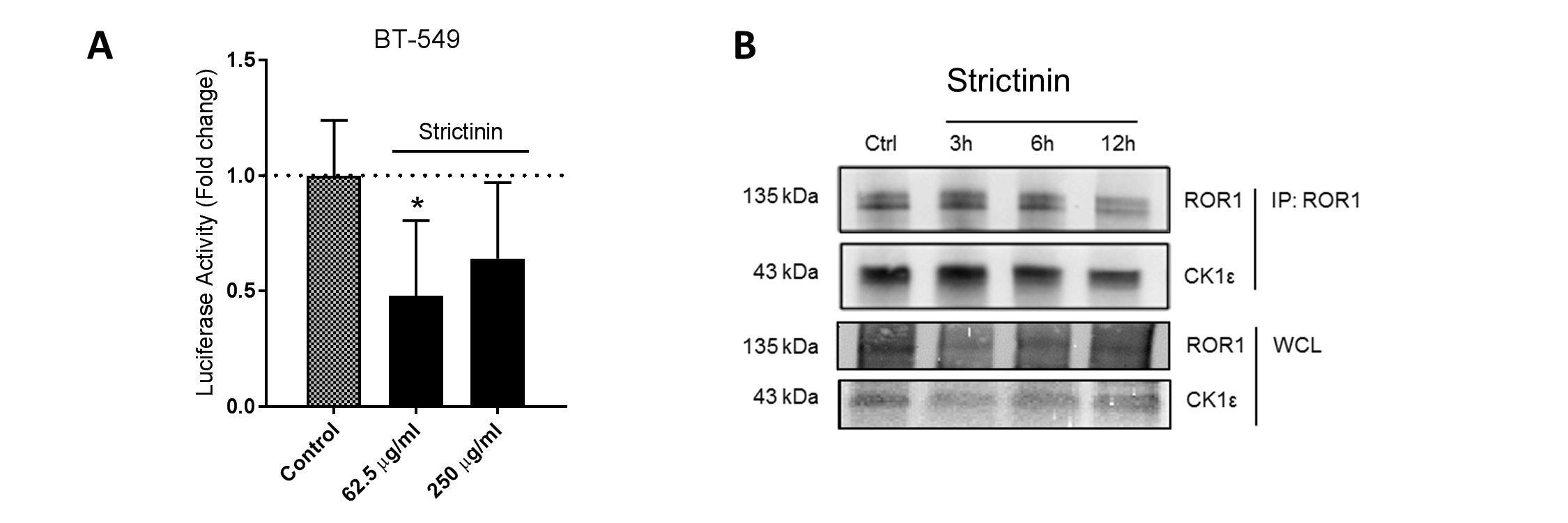

Supplement: S2 Fig — a). FOXO-luciferase assay assessing strictinin effect on P13K/AKT activity in BT-549 after 24h. (* = p.value < 0.05, n = 3) b) Co-Immunoprecipitation of ROR1 to assess CK1ε binding after strictinin treatment. (TIF) [file pone.0217789.s002.tif]

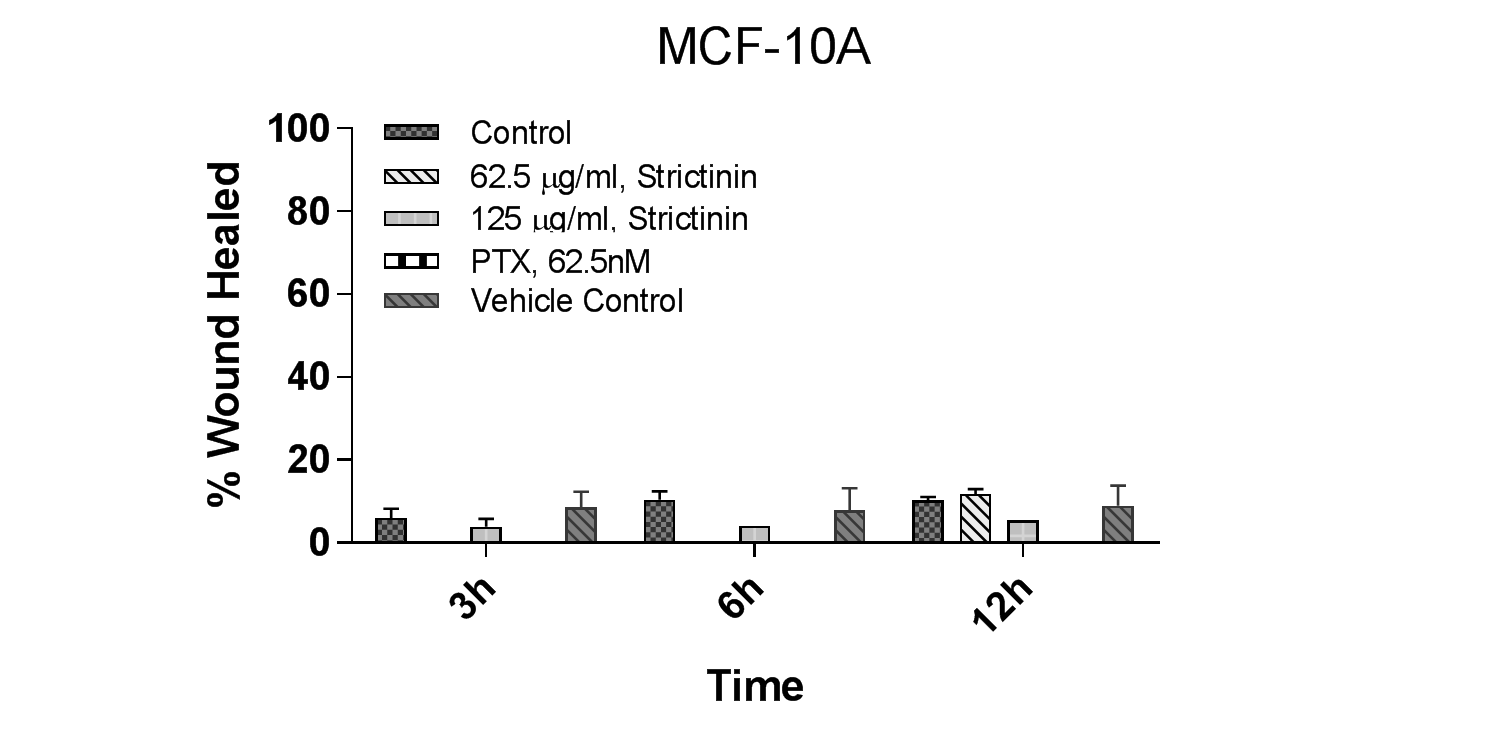

Supplement: S3 Fig — Wound healing assay investigating strictinin effect on MCF-10A migration (* = p.value < 0.05, n = 3). (TIF) [file pone.0217789.s003.tif]
